# Radiofrequency Ablation for Thyroid Nodules (RATED Study)—Analysis of a Learning Curve and Predictors of Success

**DOI:** 10.1210/clinem/dgaf058

**Published:** 2025-01-29

**Authors:** Manon M D van der Meeren, Tim Boers, Pim de Graaf, Katya M Duvivier, Koen M A Dreijerink, Laura N Deden, Peter Veendrick, Paul Cernohorsky, Frank B M Joosten, Angelique B M C Savelberg, Sicco J Braak, Sean H P P Roerink, Michel Versluis, Srirang Manohar, Wim J G Oyen

**Affiliations:** Rijnstate, Department of Radiology and Nuclear Medicine, 6815 AD Arnhem, the Netherlands; Rijnstate, Department of Internal Medicine, 6815 AD Arnhem, the Netherlands; Radboudumc, Department of Radiology and Nuclear Medicine, 6525 GA Nijmegen, the Netherlands; University of Twente, Multi-modality Medical Imaging, TechMed Center, 7522 NH Enschede, the Netherlands; Amsterdam UMC, Location Vrije Universiteit Amsterdam, Department of Radiology and Nuclear Medicine, 1081 HV Amsterdam, the Netherlands; Amsterdam UMC, Location Vrije Universiteit Amsterdam, Department of Radiology and Nuclear Medicine, 1081 HV Amsterdam, the Netherlands; Amsterdam UMC, Location Vrije Universiteit Amsterdam, Department of Endocrinology and Metabolism, 1081 HV Amsterdam, the Netherlands; Rijnstate, Department of Radiology and Nuclear Medicine, 6815 AD Arnhem, the Netherlands; Rijnstate, Department of Radiology and Nuclear Medicine, 6815 AD Arnhem, the Netherlands; Rijnstate, Department of Radiology and Nuclear Medicine, 6815 AD Arnhem, the Netherlands; Rijnstate, Department of Radiology and Nuclear Medicine, 6815 AD Arnhem, the Netherlands; Meander Medical Center, Department of Internal Medicine, 3813 TZ Amersfoort, the Netherlands; Ziekenhuisgroep Twente, Department of Radiology, 7609 PP Almelo, the Netherlands; Rijnstate, Department of Internal Medicine, 6815 AD Arnhem, the Netherlands; University of Twente, Physics of Fluids, TechMed Center, 7522 NH Enschede, the Netherlands; University of Twente, Multi-modality Medical Imaging, TechMed Center, 7522 NH Enschede, the Netherlands; Rijnstate, Department of Radiology and Nuclear Medicine, 6815 AD Arnhem, the Netherlands; Radboudumc, Department of Radiology and Nuclear Medicine, 6525 GA Nijmegen, the Netherlands; Department of Biomedical Sciences and Humanitas Clinical and Research Centre, Humanitas University, 20089 Milan, Italy

**Keywords:** thyroid, radiofrequency ablation, nodule, learning curve, ultrasound

## Abstract

**Context:**

Radiofrequency ablation (RFA) is used as treatment for symptomatic thyroid nodules. Factors influencing the volume reduction ratio (VRR) at 12 months are not yet fully understood.

**Objective:**

The primary objective of this work was evaluating the VRR at 12 months after RFA. Secondary objectives were the assessment of a learning curve and factors influencing the VRR at 12 months.

**Methods:**

A retrospective observational cohort study was conducted at 3 Dutch referral hospitals of patients who underwent RFA for symptomatic thyroid nodules with available ultrasound (US) follow-up. Main outcome measures included US-based VRR at 12 months and chronologically numbered RFA procedures. All patients’ baseline, treatment, and early follow-up factors were assessed for correlation with VRR at 12 months.

**Results:**

A total of 337 patients with 356 nodules were included in the learning curve analysis. VRR at 12 months increased for the first 20 treatments per center and stabilized thereafter, indicating a plateau phase after a learning curve. These initial cases were removed from further analysis. In the remaining 299 nodules, median VRR at 3, 6, and 12 months was 57.1%, 65.6%, and 70.8%. Baseline nodule volume negatively correlated with VRR at 12 months but VRR was high for every volume category. Energy delivered per volume did not correlate with VRR.

**Conclusion:**

In RFA for thyroid nodules, a stable treatment efficacy is achieved after 20 treatments, with a median VRR of 70.8%. Baseline nodule volume, energy delivered, and prolonged follow-up 6 months after treatment may not be clinically relevant to predict treatment success.

Since its introduction, radiofrequency ablation (RFA) for benign thyroid nodules has steadily gained interest and is today considered an option to treat nodule-related mechanical and/or cosmetic symptoms.

RFA was first described by a Korean research group in 2008 and later reported by groups in Europe and the United States (1-5). Long-term follow-up of RFA has shown that the treatment is safe and successful, reducing nodule volume by 77% 1 year after treatment (3). In 2015, RFA for benign thyroid nodules was introduced in the Netherlands, and early experiences were reported by several groups (6-8).

Several patient-, nodule-, and treatment-related factors have been studied to predict treatment outcome in terms of volume reduction ratio (VRR). Baseline nodule volume is most strongly correlated with VRR, favoring smaller nodules to achieve the highest VRRs (9). Secondly, a positive but limited correlation between energy deliverance per milliliter nodule volume has been demonstrated to ensure sufficient VRR (10). A possible issue is regrowth of the thyroid nodule after RFA, which is estimated to occur in 13% to 19% of cases, often not occurring earlier than 3 years post ablation (11, 12).

For proper implementation in clinical practice, it is critical that study results be reproduced in different populations. For RFA this validation of factors influencing VRR after RFA is limited, especially in the Netherlands, where only smaller studies have reported on RFA for benign thyroid nodules (6-8, 13, 14).

A learning curve when implementing RFA in benign thyroid nodules has been described before. Several studies demonstrate a lower VRR for the initial procedures, followed by a higher VRR in patients treated by experienced physicians in a so-called “plateau-phase.” Researchers explain this observation by increased technique proficiency over time. The number of patients treated to reach this plateau phase varies widely in studies, ranging from 20 to 90 procedures (6, 13, 15, 16). Moreover, this learning curve may be affected by other factors that influence outcome of VRR, such as nodule size, procedure complexity, and nodule composition. These influences have been scarcely investigated (15-17).

In this large retrospective study, we assessed RFA in a large Dutch cohort, using the Dutch multicenter RFA registry (RATED). The present research focused on 1, determining the effectiveness of RFA in a Dutch population; 2, the effect of the learning curve on treatment effectiveness; and 3, the contribution of ultrasound (US) characteristics, treatment parameters, and early-treatment effects to the prediction of VRR 12 months after treatment.

## Materials and Methods

### Study Design

This retrospective cohort study was performed in 3 Dutch hospitals: Amsterdam UMC, at location Vrije Universiteit (Amsterdam), Rijnstate (Arnhem), and Ziekenhuisgroep Twente (Almelo). These centers will be referred to as VUMC, RST, and ZGT, respectively. Institutional review board approval was obtained at all centers. Patient consent was waived in RST, while VUMC and ZGT used an opt-out procedure.

All patients who underwent RFA for benign thyroid nodules between July 2015 until September 2022 were eligible for inclusion.

Results of the first 103 patients of RST have been reported before, including an analysis on the effect of RFA on thyroid function in a small cohort of autonomously functioning nodules (6, 18). Since the aims of this study are different, and longer follow-up has become available, these patients were included in this study as well.

#### Standard clinical practice

Thyroid US evaluation before treatment was routinely performed. Volumes were determined using standard caliper measurements and the ellipsoid formula:


(1) $$V=l\times h\times w\times \frac{\pi }{6},$$

where *V* is the volume, *l* is the length, *h* is the height, and *w* is the width.

The benign nature of the nodule was based on US characteristics and cytopathology results.

#### Radiofrequency ablation procedure

The ablations were performed by 8 radiologists, of whom 2 performed RFAs at VUMC, 4 at RST, and 2 at ZGT. When implementing RFA, all ablations were performed by pairs of radiologists per center. After gaining experience, ablations were performed by one radiologist who consulted another radiologist if preferred. Patients received local anesthetics (1%-2% lidocaine) and a mild sedative (oral, 7.5 mg midazolam or 10 mg oxazepam). The RFA electrodes used were predominantly monopolar, working at 480 kHz, internally cooled, with a 18G diameter and varying active zone sizes of 5 to 15 mm, all with a 360° active zone (STARmed); grounding pads were placed on the shoulders or thighs. RFA was performed according to the moving-shot-technique with a transisthmic approach (19). RFA was guided by high-frequency linear US probes with working frequencies of 11 to 18MHz. RFA power setting started at 40W and was increased or decreased at the discretion of the treating physician. Per ablation session two goals were set: to ablate the nodule volume to the largest extent as possible (while preventing damage to the recurrent laryngeal nerve, blood vessels, trachea, and esophagus) and to reach a minimum total amount of energy delivered of 2.11 kJ (0.5 kcal) per milliliter (10).

After treatment, patients were observed for 1 to 3 hours (depending on treatment center) and discharged home if pain levels were acceptable (with acetaminophen, if necessary). Patients were followed in an outpatient setting at 3, 6, and 12 months after RFA. On a per-case basis additional follow-up was available (eg, multiple nodules, persistent symptoms).

#### Data collection

At baseline general and disease-specific information (age, sex, mechanical and cosmetic complaints) from the electronic patient files were recorded.

Pretreatment US nodule characteristics composition, multinodular goiter (defined as ≥2 nodules), size, components of the American College of Radiology (ACR) Thyroid Imaging, Reporting and Data System (TI-RADS) score (20), and volume were recorded. The nodules were grouped according to Mauri et al (19). Nodule composition was grouped as: solid (<10% cystic component), predominantly solid (10%-50%), predominantly cystic (50%-90%), and cystic (>90%). Volumes were divided into 5 volume categories: very small (≤2.0 mL), small (2.1-10.0 mL), medium (10.1-30.0 mL), large (30.1-60.0 mL), and very large (>60.1 mL). Additionally, to allow comparison of our results to guidelines and literature, the nodules are dichotomized in smaller and bigger than 5 cm.

The ablation parameters RF power, ablation time, total energy applied, total energy delivered, cystic component aspirated, main performing radiologist, and complications were recorded. Additionally, the energy delivered per milliliter of nodal volume was determined, and scored as successful if a target of 2.11 kJ/mL (0.5 kcal/mL) was reached (10).

Data were collected from 3, 6, and 12 months and last available follow-up visits after RFA. The VRR was calculated as follows:


(2) $$VRR=\frac{V_{bl}-V_{\;fu}\;}{V_{bl}}\times 100\text{\%},$$

where *VRR* is the volume reduction ratio, *V_fu_* is the total nodule volume at follow-up, and *V_bl_* is the total nodule volume before RFA. VRRs of more than 50% were considered a technical success, while the technical success rate is the percentage of treatments that were considered a technical success. Additionally, complications, symptoms, and US characteristics as described earlier, were recorded.

#### Major and minor complications

Side effects and complications were scored according to the proposed classification system by Mauri et al (19). Specifically, major complications known to occur after RFA were defined as damage to critical structures (eg, recurrent laryngeal nerve, vasculature, trachea, and esophagus) and full-thickness skin burns. Recurrent laryngeal nerve paralysis was scored as a major complication when confirmed by laryngoscopy. Temporary alteration of the voice was considered a side effect. Additionally, minor complications were defined as pain (requiring analgesics other than acetaminophen), swelling lasting more than 1 week, and thyroid hemorrhage. Mild pain and swelling requiring not more than acetaminophen were not recorded separately, since these are inherent to RFA treatment. Changes in thyroid function were not analyzed, since it has been well established that RFA does not negatively influence thyroid function (21).

#### Study outcomes

The primary outcome of this study was VRR at 12 months after RFA. Secondary aims were to evaluate the effect of a learning curve, factors influencing the VRR at 12 months after a possible learning curve, with special attention to the VRR at 6 months, and finally, long-term follow-up.

#### Patient selection and data analysis

Every RFA session was consecutively numbered for the benefit of the learning curve analysis. For other analyses, patients were excluded when US nodule volume data at baseline or at least 1 year follow-up (without reintervention) were missing. Patients were also excluded when the treated nodule had undergone previous RFA or ethanol ablation less than 3 months before RFA.

Data were collected using the online clinical research forms in Research Manager (Cloud9 Health Solutions). Data analysis was performed in SPSS (25, version 4, IBM). The database was locked in September 2023.

We hypothesized that 10 to 30 ablations are needed to reach a plateau phase of VRRs, based on available data in the literature (6, 13, 15, 16). Every RFA procedure was numbered consecutively, regardless of exclusion criteria, to prevent selection bias in the learning curve analysis. To determine the presence of a plateau phase and its threshold, data were grouped per 10 treatments per center and included nodules were analyzed. Groups may be unequal because of excluded patients due to previously mentioned criteria. Box plots showing the VRR at 12 months were obtained. Similarly, data per radiologist were assessed. Data were grouped for volume category and energy delivered during RFA to assess its relation to VRR at 12 months.

All data were visually checked for a normal distribution. Normally distributed data are presented as means ± SD, nonnormally distributed data as medians and interquartile range (IQR) 25th and 75th percentile in parentheses. Groups and proportions are presented as numbers and percentages. The number of missing values are reported per variable in the supplemental material (22). Means, medians, and proportions have been calculated using available data.

If the learning curve analysis showed a plateau phase, as in previous literature, the treatments before this phase were removed from the correlation analysis and the primary outcome was recalculated. Correlations were identified between variables using a Spearman rho (continuous variables) or Mann Whitney *U* or Kruskal-Wallis (categorical/ordinal variables) correlation test.

## Results

**Figure 1.**
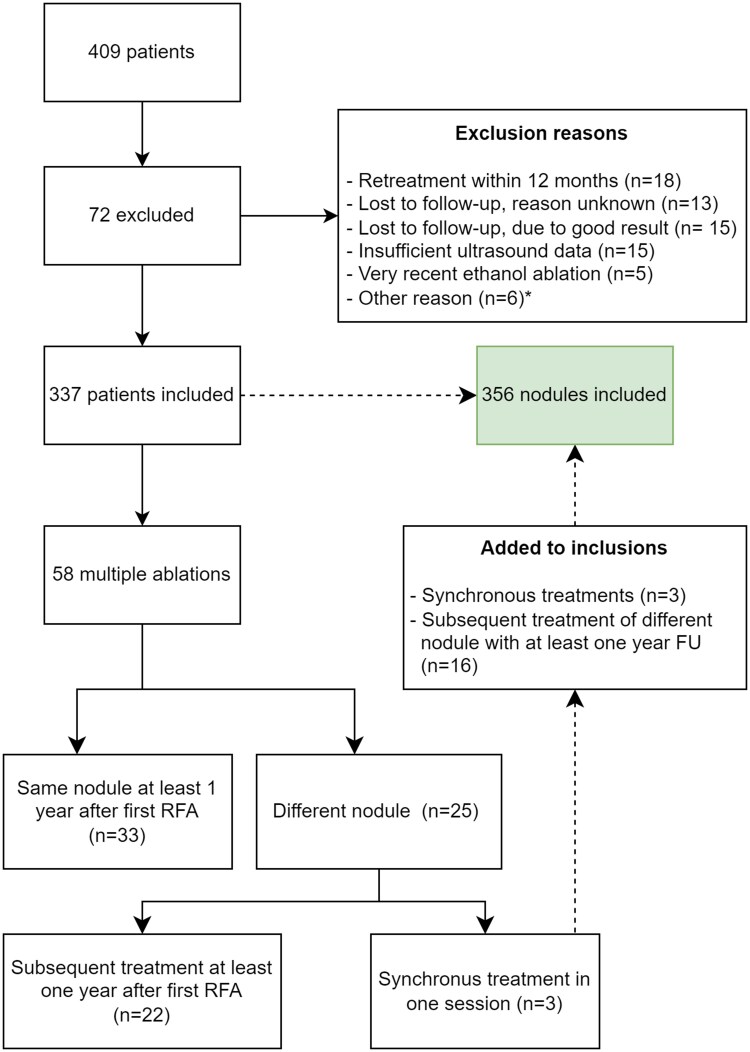
Selection process of nodules included in this study. *Lost to follow-up due to good results relates to patients that refused further follow-up as they were satisfied with their treatment. °The other reasons were 2 retreatments, 1 loss to follow-up due to comorbidities, 1 patient had a large nodule and was planned for RFA in 2 sessions, and 2 unrelated deaths.

Out of 409 patients who were treated at participating centers with RFA, 337 patients were eligible for analysis. A total of 356 nodules were included, which were treated in 353 sessions. The inclusion flowchart is shown in Fig. 1. The mean age at ablation was 51.5 ± 12.5 years, and the median baseline nodule volume was 12.2 mL (6.9-22.4 mL). Nodules were classified as very small in 15 (4.2%), small in 128 (36.0%), medium in 156 (43.8%), large in 45 (12.6%), and very large in 12 (3.4%) cases. Most nodules were completely solid or mainly solid. An overview of all characteristics is shown in Table 1. Information on data availability is presented in Supplementary Material S1 (22).

**Table 1.** Baseline population and nodule characteristics of 337 patients with 356 nodules

Characteristic	N (%)
Nodules per center	
RST	213 (59.8%)
VUMC	84 (23.6%)
ZGT	59 (16.6%)
Sex	
Female	308 (91.4%)
Male	29 (8.6%)
Age, y*^a^*	51.5 (±12.3)*^a^*
Preablation nodule volume, mL*^b^*	12.2 (6.9-22.4)*^b^*
Preablation nodule volume group, mL	
Very small, <2.0 mL	15 (4.2%)
Small, 2.1-10.0 mL	128 (36.0%)
Medium, 10.1-20.0 mL	156 (43.8%)
Large, 20.1-30.0 mL	45 (12.6%)
Very large, >30.0 mL	12 (3.4%)
Nodularity	
Multinodular goiter	247 (69.4%)
Single nodule	109 (30.6%)
Composition nodule	
Solid	116 (32.6%)
Predominantly solid	215 (60.4%)
Predominantly cystic	25 (7.0%)
Cystic	0 (0%)
Complaints	
Mechanical	298 (83.7%)
Cosmetic	65 (18.3%)

Abbreviations: RST, Rijnstate (Arnhem); VUMC, Amsterdam UMC at Vrije Universitei; ZGT, Ziekenhuisgroep Twente (Almelo).

^*a*^Mean with SD in parentheses.

^*b*^Median with 25th and 75th percentile in parentheses.

### Radiofrequency Ablation

The mean ablation time was 15.3 ± 8.3 minutes, during which a mean of 30.0 ± 18.4 kJ was introduced in the nodule, with a median power of 40W. The median amount of energy applied per volume was 2.20 kJ/mL (1.48-3.14 kJ/mL). Of the 356 ablated nodules, 173 (48.6%) reached the 2.11 kJ per mL target. Eight procedures were terminated early: One patient experienced vasovagal syncope, 3 patients experienced too much pain, 1 patient developed a stridor, and 3 patients experienced hematoma around the nodule that prevented further ablation.

No major complications occurred. A total of 19 minor complications occurred (5.3%) and consisted of pain requiring more than acetaminophen or lasting more than 1 week (n = 11), swelling lasting more than 1 week (n =2), thyroid bleeding (n = 4), bleeding at the puncture site (n = 1), and dizziness (n = 1). Side effects were reported in 12 patients (4.8%) and included 1 skin reaction to lidocaine and 11 instances of temporary hoarseness (3.1%).

### First Year Follow-up and Learning Curve

The median VRR at 3, 6, and 12 months was 55.9% (44.1%-66.0%), 65.1% (52.3%-74.7%), and 70.5% (57.8%-80.9%), respectively. Technical success was achieved in 207 (58.1%), 261 (73.3%), and 283 (79.5%) nodules at 3, 6, and 12 months.

**Figure 2.**
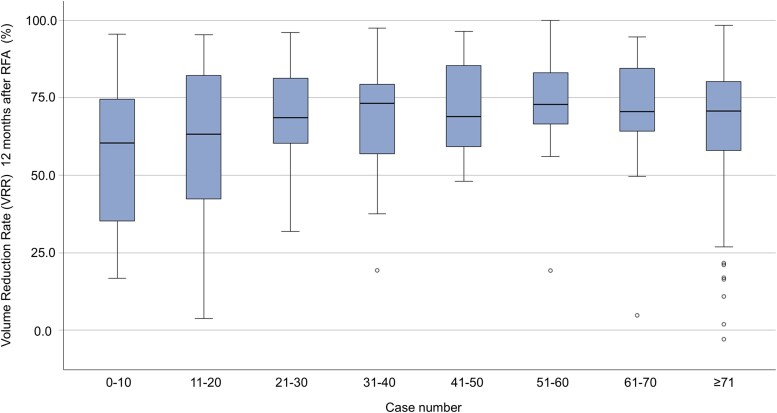
Box and whisker plot of the volume reduction ratio (VRR) at 12 months per learning curve group. An outlier with a VRR of −68% caused by a growing cystic component is not shown.

Nodule and treatment characteristics in groups of 10 consecutive treatments per center are shown in Table 2. Median VRR at 12 months is lowest in the first treatment group (60.4%) and increases per treatment group, after which it reaches a plateau after 20 treatments of approximately a median VRR of 70%. Also, the IQR of the VRR at 12 months is broadest in the first 20 treatments, and remains small after 20 treatments, indicating the plateau phase. In the final and largest group, IQR remains small, but the lowest and highest VRR have a wider spread, as shown in Fig. 2. Energy applied per milliliter increases per treatment group and baseline nodule volume remains relatively constant. Complication rates were comparable for each learning curve group.

**Table 2.** Nodule volume, volume reduction ratio at 12 months, and energy applied per case number group

Variable		Case No.							
		0-10 (n = 24)	11-20 (n = 21)	21-30 (n = 25)	31-40 (n = 26)	41-50 (n = 19)	51-60 (n = 22)	61-70 (n = 27)	70+ (n = 192)
Baseline nodule volume, mL	Median	11.6	13.3	14.1	12.3	13.6	13.4	12.2	12.0
	IQR	25.0	27.0	16.6	23.9	29.1	24.0	17.5	12.7
Energy applied per volume, kJ/mL	Median	0.94	1.02	1.68	1.57	2.1	1.88	1.94	2.59
	IQR	1.45	1.99	0.69	1.56	1.71	1.25	1.19	1.83
Energy target success rate, %		26.7	38.5	20.0	34.6	52.9	40.9	40.0	65.4
VRR 12 mo, %	Median	60.4	63.3	68.5	73.2	68.9	72.8	70.6	70.7
	IQR	40.7	48.5	22.8	24.5	27.6	17.2	21.0	22.4

Abbreviations: IQR, interquartile range; VRR, volume reduction ratio.

Individual learning curves per radiologist show a similar trend of increasing VRR at 12 months with a narrowing IQR for 4 out of 8 radiologists (1, 2, 6, and 8; Supplementary Fig. S2) (22). Interestingly, 2 radiologists (3 and 4) show a high VRR and small IQR starting from their first ablations. Both these radiologists started ablating after the center had already performed 50 and 233 ablations, respectively.

**Figure 3.**
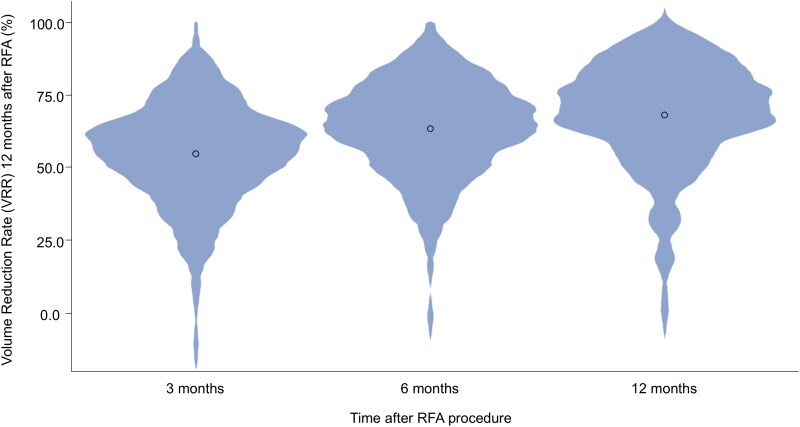
Violin plot showing distribution of VRR at 3, 6, and 12 months after radiofrequency ablation (RFA). The circle represents the mean. A trend toward a higher mean volume reduction ratio (VRR) and higher incidents of high VRRs is increasingly observed for each time point.

To account for the learning curve effect, the first 20 treatments for each center were not included in the following analysis and 311 nodules remained. The 12-month VRR was missing in 12 nodules, resulting in 299 nodules available for this analysis. In this group, median VRR at 3, 6, and 12 months was 57.1% (IQR 45.1%-66.0%), 65.6% (IQR 54.1%-74.8%), and 70.8% (IQR 59.4%-81.1%), as shown in Fig. 3. Additionally, at 12 months the VRR shows a more skewed distribution toward higher VRRs as compared to 3 and 6 months (χ^2^ = 221; *P* < .001 Friedman 2-way analysis of variance). A success rate (VRR >50%) of 61.5%, 76.3%, and 85.3% at 3, 6, and 12 months after RFA was observed.

### Parameters With Effect on Volume Reduction Rate at 12 Months

The following baseline variables correlated significantly with VRR 12 months after RFA: baseline volume (rho −0.229; *P* < .0013), baseline volume category (very small, small, medium, large, very large) (χ^2^ = 17.63; *P* = .001), largest diameter greater than 5 cm (U = −2.5; *P* = .012), and location of nodule (χ^2^ = 6.69; *P* = .035).

**Figure 4.**
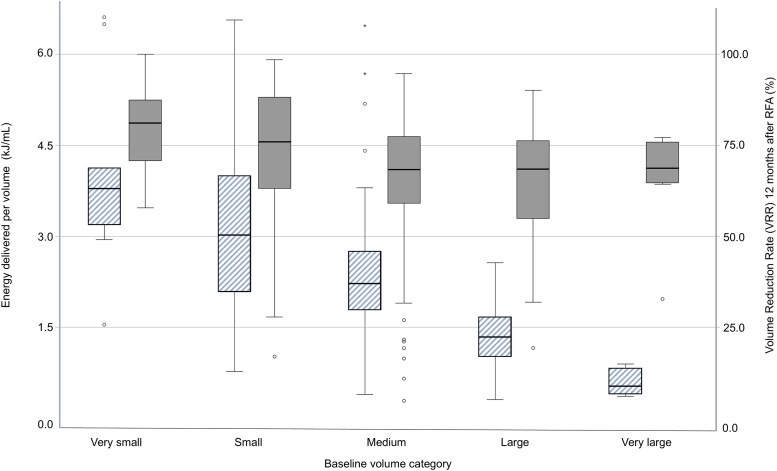
Energy delivered per volume (striped, left y-axis) and volume reduction rate at 12 months after radiofrequency ablation (filled, right y-axis) per baseline volume category (very small n = 9, small n = 111, medium n = 135, large n = 34, very large n = 10). The first 20 centers (the learning curve) have been removed. Energy delivered per volume is lower in larger baseline volume groups while volume reduction ratio (VRR) at 12 months remains relatively stable.

The VRR at 12 months was compared for the different volume and energy applied per volume (kJ/mL) categories as shown in Fig. 4 and Table 3. The amount of energy applied per milliliter strongly and significantly decreased with increasing nodule volume category (χ^2^ = 92.76; *P* < .001). The differences in VRR at 12 months for these categories, however, are small. In every volume category, median VRR at 12 months was greater than 65%. VRR at 12 months was highest in very small nodules and small nodules (81.1% and 75.9%, respectively) and lower in medium, large, and very large nodules (68.3%, 68.4%, and 68.7%, respectively). Similarly, when dividing nodules into kJ/mL categories, median baseline nodule volume decreases per kJ/mL category, with higher but similar VRR in higher kJ/mL categories. We saw no difference in nodule composition or VRR at 12 months for the different kJ/mL groups.

**Table 3.** Six cutoff groups for the amount of energy applied per volume and their relation to the volume reduction ratio at 12 months, nodule composition, and baseline nodule volume

	0.76-1.3 kJ/mL	1.3-2.1 kJ/mL	2.1-2.7 kJ/mL	<0.76 kJ/mL	>2.7 kJ/mL
	n = 31	n = 79	n = 54	n = 16	n = 107
Nodules					
Solid	11 (35.5%)	24 (30.4%)	19 (35.2%)	6 (37.5%)	34 (31.8%)
Predominantly solid	14 (45.2%)	36 (45.6%)	24 (44.4%)	7 (43.8%)	49 (45.8%)
Predominantly cystic	6 (19.4%)	19 (24.1%)	11 (20.4%)	3 (18.8%)	24 (22.4%)
Baseline volume, mL	26.9 (14.0-41.7)	16.2 (9.1-25.9)	13.8 (9.0-19.6)	40.3 (24.9-75)	8.0 (4.7-12.1)
VRR at 12 mo, %	73.7 (66.1-79.3)	68.2 (56.7-77.6)	73.8 (65.4-81.2)	67.1 (62.9-72.6)	70.7 (59.3-84.1)
Technical success at 12 mo	25 (80.8%)	64 (81.0%)	50 (92.6%)	14 (87.5%)	91 (85%)

Abbreviation: VRR, volume reduction ratio.

In conclusion, we did observe a limited relation between baseline nodule volume and VRR at 12 months, and between kJ/mL and baseline nodule volume, but not between kJ/mL and VRR 12 months.

The following follow-up variables correlated significantly to VRR at 12 months: volume at 3 months’ follow-up (rho −0.313; *P* < .001), VRR at 3 months’ follow-up (rho 0.473, *P* < .001), technical success at 3 months’ follow-up (U = 8.76; *P* < .001), volume at 6 months’ follow-up (rho −0.394; *P* < .001), VRR at 6 months’ follow-up (rho 0.591; *P* < .001), and technical success at 6 months’ follow-up (U = 9.08; *P* < .001).

The technical success rate at 6 months was 76.3% (21 missing); this increased to 85.3% at 12 months. In the 50 nodules without success at 6 months, success was reached in 17 (34%) patients at 12 months but was not achieved in the other 33 (66%) nodules. In the 228 nodules with success, 6 (2.6%) showed no success at 12 months. However, in 2 of these patients the decrease of VRR between measuring points was very limited (2.4% and 2.7%). Thus, the correct conversion from success at 6 months to no success at 12 months was observed in 4 (1.8%) nodules.

### Prolonged Follow-up and Regrowth

In 42 of 299 nodules, a prolonged follow-up after 12 months without retreatment for the ablated nodule was available. The median time to last follow-up was 26.04 months (IQR 20.8-37.5 months), at which the median VRR was 66.9% (IQR 46.0%-78.6%). The median difference between VRR at 12 months and VRR at last available follow-up was 2.8% (IQR −14.2% to 9%). Taking the measurement variability of 15% into account (23, 24), the nodule volume at last available prolonged follow-up was lower in 7 cases, higher in 15 cases, and equal in 19 cases, compared to the last available volume. Following the definition of regrowth as an increase of the total nodule volume of 50% compared to the previously recorded smallest nodule volume (19), 4 nodules showed regrowth at 12 months after RFA and an additional 7 nodules at last available follow-up. In 8 of these 11 nodules, benignancy was confirmed by repeated fine-needle aspiration or pathology diagnosis after hemithyroidectomy. Three other nodules exhibiting regrowth were toxic nodules and were subsequently treated with radioactive iodine (I131). Median baseline nodule volume and energy delivered were not statistically different for nodules expressing regrowth as compared to the total study population.

During follow-up, two malignancies were detected in an adjacent, untreated nodule. One toxic nodule, with low VRR, was shown to be malignant 12 months after follow-up.

## Discussion

This retrospective study shows a median VRR of 70.5% 12 months after treatment, with a technical success rate of 79.5%. We observed a learning curve in the first 20 patients treated per center, after which the outcome and treatment parameters stabilized (see Fig. 2 and Table 2). We showed that the baseline nodule volume and technical success at 6 months had a statistically significant predictive effect on the VRR at 12 months’ follow-up, while the much-used parameter “amount of energy applied per volume” did not (see Fig. 4).

Our results on VRR at 12 months and complications are comparable to that of the previously reported literature (3, 17). No life-threatening or severe complications occurred in our cohort.

Both the VRR and energy applied per milliliter showed an increase when plotted against the number of procedures, which is similar to earlier reports (6, 13, 15, 16). The VRR improved in the first 20 cases per center and remained relatively stable thereafter (see Fig. 2 and Table 2). We assessed the learning curve by plotting the VRR against the number of procedures, similar to the previous literature (6, 13). Others have used a CUSUM analysis (15, 16). Previous studies assessed the learning curve in 1 or 2 radiologists, whereas we present information for 3 centers with 8 radiologists. The observed learning curve in our cohort of 20 cases per center is short compared to the previous literature. This may be attributed to well-executed proctoring, thereby building on existing experience. Furthermore, the RFAs have often been performed by pairs of radiologists, thus while not actively ablating, a radiologist can still experience a learning effect (see Supplementary Fig. S2) (22).

We did not find a correlation between the VRR at 12 months and energy applied per volume, while previous studies report this as a predictive parameter both for technical and clinical success (5, 10). The previously postulated threshold may in fact be lower than presumed and is reached for most nodules in our cohort. Another explanation for why we have not found a correlation is that the applied energy per volume requirement is lower for larger nodule volumes, thus larger nodules may be ablated as effectively while applying less energy per volume (see Fig. 4 and Table 3). For example, the energy requirement may be affected by nodule composition or other unknown factors (10). Although we did not observe data that support the use of an energy target, the applied energy per volume does offer guidance in terms of treatment completion. We suggest further research to investigate the suitability of this target.

For a patient to be a candidate for RFA, different limits of nodule size and volumes have been suggested, assuming that a larger nodule may result in a worse clinical outcome. Limits include a maximum dimension of 5 cm, or a volume of 20 mL (25-27). Our data indeed show a significantly lower VRR at 12 months in larger nodules. However, the difference in treatment effect is small, even the large (30.0-60.0 mL) and very large (>60.1 mL) nodules showed a good median VRR at 12 months of 68.4% and 68.9%, respectively, and a technical success rate of 76.5% and 90%, respectively. These findings support RFA treatment of nodules larger than currently recommended in guidelines (25-27).

Our study confirms that after RFA, nodules decrease in size mostly in the first 3 to 6 months and very little between 6 to 12 months (see Fig. 3). Additionally, if technical success is achieved at 6 months, only in 1.8% of nodules is technical success not achieved at 12 months. Therefore, VRR at 6 months may be considered as a cutoff point for clinicians to decide on retreatment, continuing follow-up, or discontinuing follow-up when VRR and relief of symptoms are sufficient.

Neither our data, nor previous reports, suggest the occurrence of thyroid-specific malignancy after RFA. However, this was not the aim of this study (11, 12). When discontinuing follow-up at 6 months after RFA, the risk of developing malignancy may be considered before discharge. Baseline TI-RADS should be assessed, and patients should be instructed to return if symptoms or visible growth recur. Summarizing, the VRR at 6 months may be suitable for clinical decision-making during follow-up after RFA but should be investigated further.

This study was limited by its retrospective nature, which resulted in incomplete data (Supplementary Table S1) (22). There may also be selection bias, due to selecting patients with US data available at 12 months after RFA. Both overestimation and underestimation of VRR at 12 months may have occurred due to cases that were not analyzed (see Fig 1). A similar number of patients discontinued follow-up because of good clinical effect compared to the number early retreatments due to insufficient clinical effect. An additional 13 patients stopped follow-up for unknown reasons, but having a sufficient treatment effect seems most likely, as the patients did not return with thyroid nodule symptoms. For the other 26 excluded patients, treatment result is more difficult to predict. Overall, given the remaining large sample size, we do not expect that the effect of missing data resulted in a significant bias of our result. Also, our information about follow-up after the first year is limited, since information from only 42 of 299 patients was available. Since this study was conducted in the Netherlands, we predominantly included Dutch patients, which may limit the generalizability to populations with a different distribution regarding population ethnicity.

Another limitation of this and previous reports is the scarce data on clinical success, for example, symptom relief or thyroid-related quality of life. At present, the effect of RFA is usually expressed in VRR. The commonly used cutoff for technical success at greater than 50% has, to our knowledge, not yet been correlated to clinical outcomes such as symptom relief. Future studies should evaluate this relationship to set meaningful treatment goals.

## Conclusion

With a 12-month VRR of 70.5% and a success rate (VRR >50%) of 79.5%, RFA in 3 Dutch hospitals was found to be effective in reducing thyroid nodule volume in 356 nodules. A learning curve was observed for the first 20 cases, after which median VRR at 12 months increased from 60.4% in the first cases, to 70.8% after removal of the first cases. VRR at 12 months was highest in the smallest nodules; still, median VRR at 12 months was greater than 68% in every volume category. Energy delivered per volume did not affect the VRR. The VRR remains stable between 6 and 12 months, and future studies can assess its suitability as an end point for follow-up and its relation to clinical success.

## Data Availability

Some or all data sets generated during and/or analyzed during the present study are not publicly available but are available from the corresponding author on reasonable request.

## Abbreviations

IQR: interquartile range
RFA: radiofrequency ablation
RST: Rijnstate (Arnhem)
TI-RADS: Thyroid Imaging, Reporting and Data System
US: ultrasound
VRR: volume reduction ratio
VUMC: Amsterdam UMC at Vrije Universitei
ZGT: Ziekenhuisgroep Twente (Almelo)

## References

[1] Jung SL, Baek JH, Lee JH, et al Efficacy and safety of radiofrequency ablation for benign thyroid nodules: a prospective multicenter study. Korean J Radiol. 2018;19(1):167‐174. 29354014 10.3348/kjr.2018.19.1.167 PMC5768499

[2] Dobnig H, Amrein K. Monopolar radiofrequency ablation of thyroid nodules: a prospective Austrian single-center study. Thyroid. 2018;28(4):472‐480. 29490593 10.1089/thy.2017.0547 PMC5905420

[3] Cho SJ, Baek JH, Chung SR, Choi YJ, Lee JH. Long-term results of thermal ablation of benign thyroid nodules: a systematic review and meta-analysis. Endocrinol Metab (Seoul). 2020;35(2):339‐350. 32615718 10.3803/EnM.2020.35.2.339 PMC7386110

[4] Chen F, Tian G, Kong D, Zhong L, Jiang T. Radiofrequency ablation for treatment of benign thyroid nodules: a PRISMA-compliant systematic review and meta-analysis of outcomes. Medicine (Baltimore). 2016;95(34):e4659. 27559968 10.1097/MD.0000000000004659 PMC5400335

[5] Deandrea M, Garino F, Alberto M, et al Radiofrequency ablation for benign thyroid nodules according to different ultrasound features: an Italian multicentre prospective study. Eur J Endocrinol. 2019;180(1):79‐87. 30407921 10.1530/EJE-18-0685

[6] Bom WJ, Joosten FBM, van Borren MMGJ, Bom EP, van Eekeren RRJP, de Boer H. Radiofrequency ablation for symptomatic, non-functioning, thyroid nodules: a single-center learning curve. Endocr Connect. 2022;11(1):e210304. 34887358 10.1530/EC-21-0304 PMC8859967

[7] Lončar I, van Dijk SPJ, van Velsen EFS, et al Radiofrequency ablation for benign symptomatic thyroid nodules in The Netherlands: successful Introduction of a minimally invasive treatment option improving quality of life. J Vasc Interv Radiol. 2022;33(5):530‐537.e1. 35121096 10.1016/j.jvir.2022.01.012

[8] van Baardewijk LJ, Plaisier ML, van den Broek FJC, van Poppel PCMW, Kurban S, Kruimer JWH. Tracheal necrosis following radiofrequency ablation of a benign thyroid nodule. Cardiovasc Intervent Radiol. 2021;44(1):170‐171. 32909063 10.1007/s00270-020-02632-0

[9] Cesareo R, Naciu AM, Iozzino M, et al Nodule size as predictive factor of efficacy of radiofrequency ablation in treating autonomously functioning thyroid nodules. Int J Hyperthermia. 2018;34(5):617‐623. 29357717 10.1080/02656736.2018.1430868

[10] Deandrea M, Trimboli P, Mormile A, et al Determining an energy threshold for optimal volume reduction of benign thyroid nodules treated by radiofrequency ablation. Eur Radiol. 2021;31(7):5189‐5197. 33409792 10.1007/s00330-020-07532-y

[11] Yan L, Zhang M, Li X, Li Y, Luo Y. A nomogram to predict regrowth after ultrasound-guided radiofrequency ablation for benign thyroid nodules. Front Endocrinol (Lausanne). 2021;12:774228. 35250847 10.3389/fendo.2021.774228 PMC8891142

[12] Li Y, Li W, Jiang B, Zhao J, Zhang Y, Luo Y. Analysis and prediction of regrowth in benign thyroid nodules undergoing radiofrequency ablation: a retrospective study with a 5-year follow-up. Eur Radiol. 2023;33(8):5615‐5624. 36951983 10.1007/s00330-023-09481-8

[13] Russ G, Ben Hamou A, Poirée S, et al Learning curve for radiofrequency ablation of benign thyroid nodules. Int J Hyperthermia. 2021;38(1):55‐64. 33491515 10.1080/02656736.2021.1871974

[14] Motaghed Z, Chegeni H, Mosadeghkhah A, Azimi Aval M, Gerami R, Ebrahiminik H. Effect of ultrasound parameters of benign thyroid nodules on radiofrequency ablation efficacy. BMC Med Imaging. 2023;23(1):85. 37337132 10.1186/s12880-023-01044-z PMC10278342

[15] Kuo CY, Liu CL, Tsai CH, Cheng SP. Learning curve analysis of radiofrequency ablation for benign thyroid nodules. Int J Hyperthermia. 2021;38(1):1536‐1540. 34727824 10.1080/02656736.2021.1993358

[16] Chytiris S, Teliti M, Croce L, et al Proficiency in performing radiofrequency ablation procedure for non-functioning benign thyroid nodules: a qualitative rather than quantitative matter. Front Endocrinol (Lausanne). 2024;15:1399912. 38933827 10.3389/fendo.2024.1399912 PMC11200030

[17] Trimboli P, Castellana M, Sconfienza LM, et al Efficacy of thermal ablation in benign non-functioning solid thyroid nodule: a systematic review and meta-analysis. Endocrine. 2020;67(1):35‐43. 31327158 10.1007/s12020-019-02019-3

[18] van der Meeren MMD, Joosten FBM, Roerink SHPP, Deden LN, Oyen WJG. Radiofrequency ablation for autonomously functioning nodules as treatment for hyperthyroidism: subgroup analysis of toxic adenoma and multinodular goitre and predictors for treatment success. Eur J Nucl Med Mol Imaging. 2023;50(12):3675‐3683. 37466647 10.1007/s00259-023-06319-9 PMC10547644

[19] Mauri G, Pacella CM, Papini E, et al Image-guided thyroid ablation: proposal for standardization of terminology and reporting criteria. Thyroid. 2019;29(5):611‐618. 30803397 10.1089/thy.2018.0604

[20] Tessler FN, Middleton WD, Grant EG, et al ACR thyroid imaging, reporting and data system (TI-RADS): white paper of the ACR TI-RADS committee. J Am Coll Radiol. 2017;14(5):587‐595. 28372962 10.1016/j.jacr.2017.01.046

[21] Nixon IJ, Angelos P, Shaha AR, Rinaldo A, Williams MD, Ferlito A. Image-guided chemical and thermal ablations for thyroid disease: review of efficacy and complications. Head Neck. 2018;40(9):2103‐2115. 29684251 10.1002/hed.25181

[22] van der Meeren MMD, Boers T, de Graaf P, et al Data underlying the publication: radiofrequency ablation for thyroid nodules (RATED study)—analysis of a learning curve and predictors of success. *4TU.ResearchData*. 2025. Doi: 10.4121/dd66122a-77c2-4f05-a34a-6b5aed8223e0 PMC12527443 39880382

[23] Choi YJ, Baek JH, Hong MJ, Lee JH. Inter-observer variation in ultrasound measurement of the volume and diameter of thyroid nodules. Korean J Radiol. 2015;16(3):560‐565. 25995685 10.3348/kjr.2015.16.3.560 PMC4435986

[24] Lee HJ, Yoon DY, Seo YL, et al Intraobserver and interobserver variability in ultrasound measurements of thyroid nodules. J Ultrasound Med. 2018;37(1):173‐178. 28736947 10.1002/jum.14316

[25] Papini E, Monpeyssen H, Frasoldati A, Hegedüs L. 2020 European thyroid association clinical practice guideline for the use of image-guided ablation in benign thyroid nodules. Eur Thyroid J. 2020;9(4):172‐185. 32903999 10.1159/000508484 PMC7445670

[26] Kim JH, Baek JH, Lim HK, et al 2017 thyroid radiofrequency ablation guideline: korean society of thyroid radiology. Korean J Radiol. 2018;19(4):632‐655. 29962870 10.3348/kjr.2018.19.4.632 PMC6005940

[27] Association. DE . Schildkliernodus en struma. 2017. Accessed July 4, 2024. https://www.nve.nl/aandoening/schildkliernodus/

